# The risk of developing a meningioma during and after pregnancy

**DOI:** 10.1038/s41598-021-88742-2

**Published:** 2021-04-28

**Authors:** Jenny Pettersson-Segerlind, Tiit Mathiesen, Adrian Elmi-Terander, Erik Edström, Mats Talbäck, Maria Feychting, Giorgio Tettamanti

**Affiliations:** 1 Department of Neurosurgery, Karolinska University Hospital, Stockholm, Sweden; 2 Department of Clinical Neuroscience, Karolinska Institutet, Stockholm, Sweden; 3 Department of Neurosurgery, Rigshospitalet, Copenhagen, Denmark; 4 Institute of Clinical Medicine, University of Copenhagen, Copenhagen, Denmark; 5 Unit of Epidemiology, Institute of Environmental Medicine, Karolinska Institutet, Stockholm, Sweden

**Keywords:** Cancer epidemiology, CNS cancer

## Abstract

Pregnancy has been associated with diagnosis or growth of meningiomas in several case reports, which has led to the hypothesis that pregnancy may be a risk factor for meningiomas. The aim of this study was to test this hypothesis in a large population-based cohort study. Women born in Sweden 1958–2000 (N = 2,204,126) were identified and matched with the Medical Birth Register and the Cancer Register. The expected number of meningioma cases and risk ratios were calculated for parous and nulliparous women and compared to the observed number of cases. Compared to parous women, meningiomas were more common among nulliparous (SIR = 1.73; 95% CI 1.52–1.95). The number of meningioma cases detected during pregnancy was lower than the expected (SIR = 0.40; 95% CI 0.20–0.72). Moreover, no increased risk was found in the first-year post-partum (SIR = 1.04; 95% CI 0.74–1.41). Contrary to our hypothesis, there was no increased risk for diagnosing a meningioma during pregnancy or 1-year post-partum. A lower detection rate during pregnancy, may reflect under-utilization of diagnostic procedures, but the actual number of meningiomas was homogenously lower among parous than nulliparous women throughout the study period, indicating that pregnancy is not a risk factor for meningioma.

## Introduction

Meningiomas are the largest group of primary intracranial and intraspinal tumors, comprising approximately one third of the tumors^1,2^. One key factor in meningioma epidemiology is the relation to sex since two thirds of the tumors occur in women, while the only other established risk factors for meningioma are increasing age, exposure to ionizing radiation, and some rare genetic syndromes. The incidence of meningioma has increased among fertile and menopausal women during the last 30 years while the incidence among men has been stationary^3^. Epidemiological studies on the association between meningiomas and exogenous sex-hormone treatment have shown conflicting results^4–7^. Post-menopausal hormone replacement therapy that exposes women to elevated levels of sex hormones increased the risk for developing a meningioma in several studies^6,8–10^, while others failed to find an association^11–13^. Recently, a French nationwide epidemiological study showed a strong correlation between hormone therapy with cyproterone acetate, a synthetic antiandrogen and progestagen, and a meningioma diagnosis^14^. Taken together, the sex distribution and empirical observations of biological and epidemiological associations between sex hormones and meningiomas warrant further exploration.

Traditional neurosurgical literature considered pregnancy to be associated with meningiomas^15,16^. Dramatic changes of progesterone, estradiol, testosterone and prolactin levels occur during pregnancy^17^. Progesterone, estrogen and their metabolites, increase during pregnancy and reach the highest levels in the third trimester. Progesterone increases approximately 6–8 times compared to a normal menstrual cycle, while estrogen rises 15–75 times compared to luteal phase and 60–300 times compared to the follicular phase in a normal menstrual cycle^18^. Pregnancy, with its extensive hormonal changes, could be a risk factor for meningioma development or growth given the epidemiological associations with hormone levels. Most meningiomas express receptors for progesterone but not estrogen^19^ and the hormonal effects depend on a balance between estrogen and progesterone^20^. Progesterone signaling is mitogenic in breast cancer^21^ and progesterone increases sensitivity of meningioma cells to mitogenic stimuli^22^. In agreement with this hypothesis, cases of rapid meningioma growth during pregnancy, sometimes even followed by spontaneous involution post-partum, have been described since meningiomas were first classified^23–25^. The evidence for an association between meningiomas and pregnancy has been anecdotal and epidemiological validation is scarce. Wigertz et al. found a correlation between the risk of a meningioma and three or more live births among women younger than 50 years, in a case-control study^5^, while other studies have been negative^10,13^.

In this population-based cohort study, the aim was to study the relation between giving birth and meningiomas in women during the fertile and reproductive years, specifically the risk of having a meningioma diagnosed during pregnancy and during the first 20 years postpartum, as well as the risk of meningioma among nulliparous women.

## Materials and methods

All women born in Sweden between 1958 and 2000, were identified using the national population register (Population Register) held by the Swedish national statistical bureau (Statistics Sweden) (n = 2,204,126). To make the information about childbearing as accurate as possible, women who had once emigrated (n = 203,435; 9.2%) and those lost to follow-up (n = 296) were excluded. Women who had been diagnosed with a meningioma (n = 24) or had died (n = 14,891; 0.7%) before the age of 15, were also excluded. The final cohort consisted of 1,985,480 women. Through the unique personal identity number assigned to all residents in Sweden, the cohort was linked to the national birth register (Swedish Medical Birth Register), to identify all births given by women in the cohort, from 15 years of age until the end of the study period, on December 31st, 2015. Thus, during the study, women in the cohort would be between 15 and 57 years old. The national birth register includes all births given in Sweden from 1973 onwards and an estimate of the length of each pregnancy, which was used to calculate the time of conception. Cases of meningioma were identified through the national cancer register (Swedish Cancer Register), which was initiated in 1958 with mandatory reporting of cases by both clinicians and pathologists^26^. Information about the date of death was retrieved from the national cause of death register (Swedish Cause of Death Register)^27^. The date of diagnosis in the cancer register often corresponds to the date of the pathology report, i.e. after surgery. To avoid potential bias from delayed surgery among pregnant women until after delivery, additional information regarding the date of diagnosis of meningioma, was collected from the national patient register (National Patient Register), where all hospitalizations are registered^28^. The earliest date of diagnosis in either the cancer or the patient registry was used as date of diagnosis. The three latter registers are held by the Swedish National Board of Health and Welfare.

The women in the cohort were followed from the month they turned 15 years old to the date of meningioma diagnosis, death or until December 31st, 2015. The follow-up time in days for each woman, was split into: (1) the time prior to first conception, (2) the time during pregnancy, and (3) the time between delivery and the woman’s next conception or end of follow-up. Steps 2 and 3 were repeated for each pregnancy a woman had gone through. After the data had been split, the number of cases and person-years at risk were aggregated over the covariates that were included in each respective analysis.

Based on the assumption that there was no difference between the groups, the expected number of meningioma cases during pregnancy and during the first year immediately following delivery, was calculated using age and calendar year specific incidence rates for the entire cohort. In addition, the expected number of meningioma cases among women who were nulliparous during the whole study period was calculated based on the age- and calendar year specific incidence rates among women who gave birth at any time during the follow-up. The expected number of deaths in the nulliparous group was calculated in the same way.

The expected number of cases and standardized incidence ratios (SIR) were calculated using indirect standardization. Confidence intervals for the expected number of cases were calculated assuming the theoretical Poisson distribution. Meningioma incidence rate ratios (IRR) during pregnancy and for 1 to 20 years following last delivery were estimated with a generalized linear model using a Poisson distribution with a log link, with the number of cases as the dependent variable and person-years at risk as the number exposed. In a sensitivity analysis, women with a phacomatosis (genetic syndrome associated with the occurrence of central nervous system tumors) were excluded. The correlation between attained age and time since last delivery was estimated with Pearson correlation. Statistical analyses were conducted using Stata version 14.2 (StataCorp LLC College Station, Texas, USA). The study was approved by the Regional Ethical Review Board in Stockholm (dnr 2011/634-31/4, 2016/27-32, and 2018/1257-32). All research was performed in accordance with the relevant guidelines and regulations and informed consent from study participants was not required according to local legislation.

### Ethical approval

The study was approved by the Regional Ethical Review Board in Stockholm (dnr 2011/634-31/4, 2016/27-32, and 2018/1257-32).

## Results

During the follow-up period 1973–2015 there were 2,353,894 births, and 1,129,484 women had at least one delivery, 1173 women were diagnosed with meningioma, and 21,953 women died during follow-up. Descriptive characteristics of the women included into the study are reported in Table 1. In total, the cohort accrued 42,794,231 person-years of follow-up (Table 2). Eleven women were diagnosed with meningioma during pregnancy, compared to 27.4 expected (95% CI 17.8–38.1) if the incidence during pregnancy had been the same as the incidence in the entire cohort 1973–2015 (SIR 0.40; 95% CI 0.20–0.72). During the first year immediately following delivery, 40 meningioma cases were diagnosed, compared to 38.6 expected (95% CI 26.9–51.0, SIR 1.04; 95% CI 0.74–1.41). Of these 40 women, 6 (15%) were diagnosed within the first 3 weeks after delivery, 6 (15%) between > 3 weeks and 3 months, and 11 (28%) between > 3 and 6 months. Thus, 58% were diagnosed within 6 months after delivery.

**Table 1:** Descriptive characteristics of the women included into the study.

	N of children at end of follow-up				
	0	1	2	3	4+
	N (%)	N (%)	N (%)	N (%)	N (%)
**Birth decade**					
1958–1969	83,330 (14)	84,901 (15)	257,854 (44)	118,624 (20)	40,253 (7)
1970–1979	68,080 (16)	72,029 (16)	207,053 (47)	73,159 (17)	19,022 (4)
1980–1989	207,108 (48)	94,388 (22)	104,152 (24)	23,334 (5)	4796 (1)
1990–2000	497,478 (94)	22,289 (4)	6886 (1)	660 (0)	84 (0)
**Age at first childbirth**					
Nulliparous	855,996 (100)	–	–	–	–
< 20	–	12,285 (15)	26,453 (32)	25,674 (31)	19,117 (23)
20–24	–	57,292 (16)	164,962 (47)	93,458 (27)	32,064 (9)
25–29	–	89,683 (22)	237,398 (58)	73,542 (18)	11,033 (3)
30–34	–	72,987 (33)	123,938 (56)	21,376 (10)	1850 (1)
35+	–	41,360 (62)	23,194 (35)	1727 (3)	91 (0)

The percentages reported in this table are row percentages.

**Table 2:** Person-years of follow-up and the number of births, deaths, and meningioma tumors in 5-year age groups, respectively.

Age^a^	Women with at least one delivery				Women without children		
	Number of			Person-yr.’s	Number of		Person-yr.’s
	Births^b^	Deaths	Tumors		Deaths	Tumors	
15–19	55,129	15	1	5,638,765	2219	22	3,756,930
20–24	443,253	225	6	5,598,316	2341	29	2,613,812
25–29	822,820	631	32	5,378,902	1689	41	1,520,173
30–34	700,281	1117	86	4,870,679	1279	37	937,310
35–39	280,886	1633	165	4,121,828	1034	35	673,416
40–44	49,509	2040	228	3,203,002	928	43	503,164
45–49	1965	2299	242	2,173,442	964	38	345,030
50+	51	2635	149	1,256,686	904	19	202,776
All ages	2,353,894	10,595	909	32,241,620	11,358	264	10,552,611

Women with at least one delivery and women without children during the follow-up period 1973–2015.

^a^Attained age at delivery, death or tumor diagnosis.

^b^Multiple births are counted as one birth.

**Figure 1:**
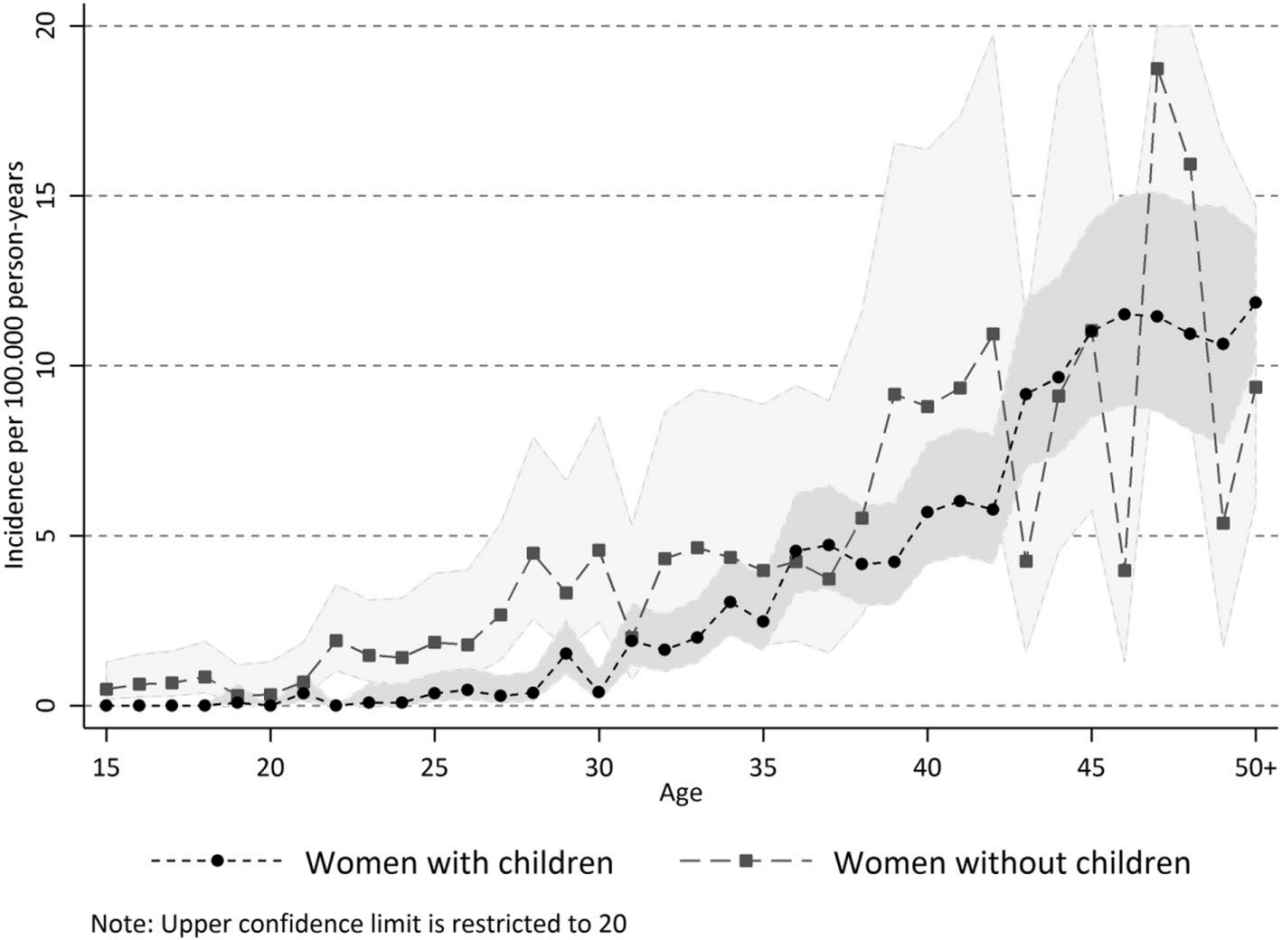
Age-specific incidence of meningioma per 100.000 person-years, stratified on women who have given birth after they turned 15 years old, and women who remained nulliparous during the follow-up period 1973–2015. Figure created using Stata version 14.2 (StataCorp LLC College Station, Texas, USA, www.stata.com).

Women who had never given birth to a child had a higher incidence of meningioma compared to parous women. Among women who were nulliparous throughout the whole follow-up period (n = 855,996), 264 were diagnosed with meningioma compared to an expected number of 153.1 (95% CI 129.7–178.2) had they had the same incidence as women who had given birth at any time during the follow-up period (SIR 1.73; 95% CI 1.52–1.95). Figure 1 shows age specific incidence rates with 95% confidence intervals for these two groups of women separately. The higher incidence in the nulliparous group seem to be primarily before age 40, although the small number of women in the older age-groups makes incidence estimates unstable. Of note is also that the overall mortality was higher in women without children, with 11,358 observed deaths compared to 1891 (95% CI 1806–1976) that would have been expected if they had had the same mortality as women who had given birth (SMR 6.01; 95% CI 5.90–6.12).

**Figure 2:**
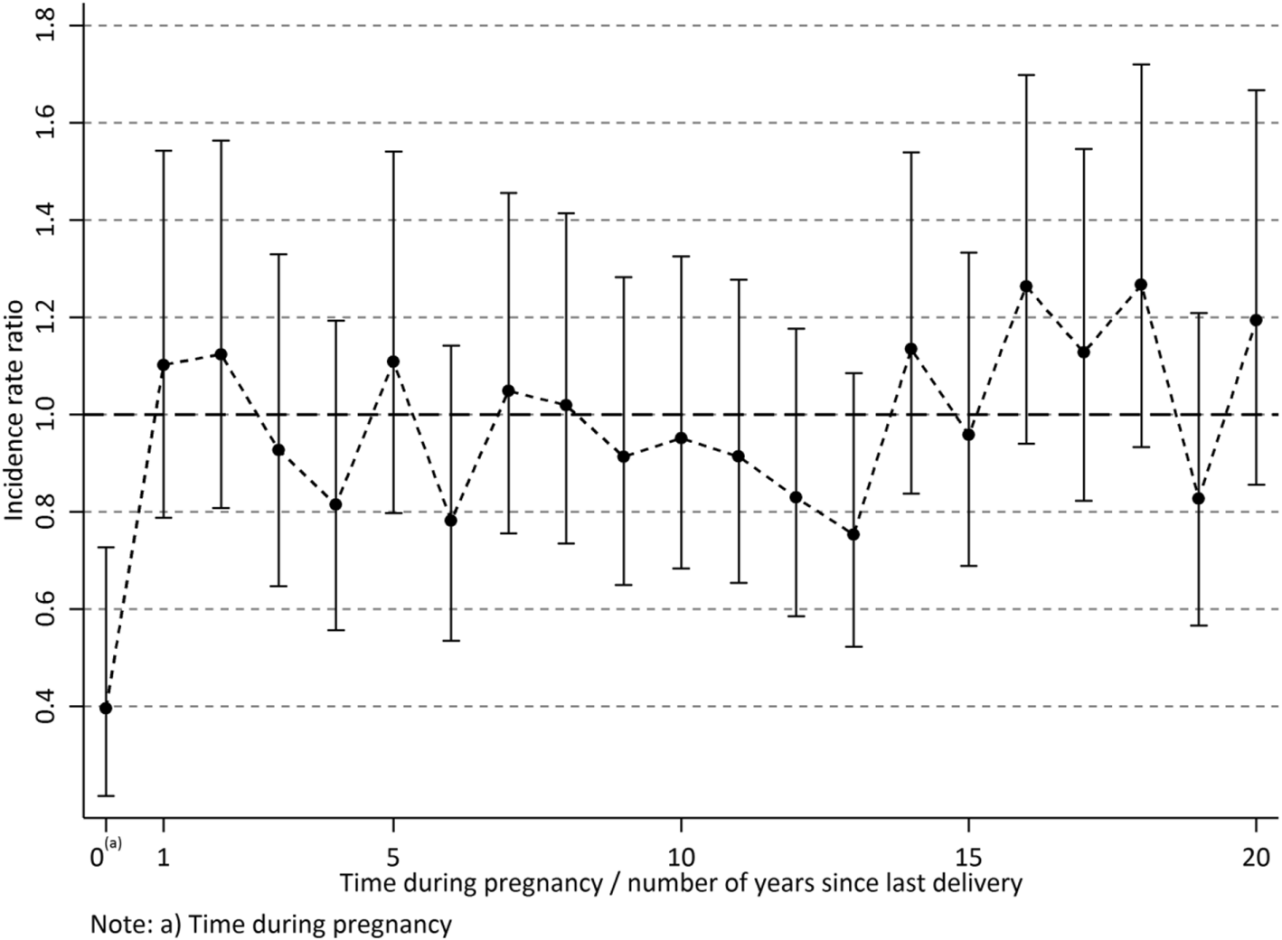
Incidence rate ratio with 95% confidence intervals adjusted for attained age by time during pregnancy/number of years since last pregnancy. Women with at least one delivery during the follow-up period 1973–2015. Figure created using Stata version 14.2 (StataCorp LLC College Station, Texas, USA, www.stata.com).

Table 3 shows IRRs for meningioma per time since last delivery, restricted to the population of women who gave birth at least once after they turned 15 years. There was a strong correlation between attained age and time since last delivery (Pearson 0.81; 95% CI 0.79–0.83). Age-adjusted IRRs show that during pregnancy the incidence of meningioma was reduced (IRR 0.40; 95% CI 0.22–0.73), while the IRR 2 years after delivery was only slightly raised, and not statistically significant. No consistent pattern with longer times since last delivery was found when adjusting for age, as displayed also in Fig. 2. Sensitivity analyses conducted after having excluded women with phacomatoses (N = 1276) showed almost identical results (data not shown).

**Table 3:** Number of meningioma tumors diagnosed during pregnancy and number of years since last delivery by delivery number.

Year^a^	No. of cases according to number of previous deliveries				Total	Incidence rate ratio^c^					
	1	2	3	4+		Unadjusted			Adjusted^d^		
						IRR	95% CI		IRR	95% CI	
0^b^	2	4	5	0	11	0.12	0.07	0.22	0.40	0.22	0.73
1	11	15	9	5	40	0.35	0.25	0.48	1.10	0.79	1.54
2	12	16	7	5	40	0.44	0.32	0.61	1.12	0.81	1.56
3	7	13	9	3	32	0.46	0.32	0.65	0.93	0.65	1.33
4	6	14	6	2	28	0.49	0.33	0.71	0.81	0.56	1.19
5	12	16	8	2	38	0.77	0.56	1.07	1.11	0.80	1.54
6	5	13	8	2	28	0.63	0.43	0.92	0.78	0.53	1.14
7	5	22	8	3	38	0.96	0.69	1.32	1.05	0.76	1.46
8	7	21	6	4	38	1.04	0.75	1.44	1.02	0.73	1.41
9	4	16	12	3	35	1.04	0.74	1.46	0.91	0.65	1.28
10	8	16	10	3	37	1.19	0.86	1.65	0.95	0.68	1.32
11	7	19	8	2	36	1.24	0.89	1.74	0.91	0.65	1.28
12	4	23	5	1	33	1.22	0.86	1.73	0.83	0.59	1.18
13	5	17	7	1	30	1.19	0.83	1.72	0.75	0.52	1.08
14	7	24	11	2	44	1.91	1.41	2.59	1.13	0.84	1.54
15	6	18	11	2	37	1.72	1.24	2.39	0.96	0.69	1.33
16	5	30	9	3	47	2.38	1.78	3.20	1.26	0.94	1.70
17	8	18	13	2	41	2.24	1.64	3.06	1.13	0.82	1.55
18	6	25	10	3	44	2.62	1.94	3.55	1.27	0.93	1.72
19	7	12	8	1	28	1.79	1.23	2.61	0.83	0.57	1.21
20	9	21	6	1	37	2.66	1.91	3.69	1.19	0.85	1.67
21+	41	89	28	9	167	3.00	2.53	3.55	1.21	0.97	1.50
Total	184	462	204	59	909	–	–	–	–	–	–

Unadjusted and adjusted incidence rate ratio (IRR) with 95% confidence intervals by number of years since last delivery.

Women with at least one delivery during the follow-up period 1973–2015.

–, Not applicable.

^a^Number of years since last delivery.

^b^Time during pregnancy.

^c^Incidence rate ratio for number of years since last delivery versus all other years.

^d^Adjusted for attained age.

## Discussion

Given the available literature on differences in meningioma incidence related to sex, hormonal therapy and pregnancy, we had expected to detect an increased incidence of meningiomas associated with pregnancy in this population-based cohort study^1,2,4–13,24,25^. However, contrary to our expectations, we found a lower incidence of meningiomas among women who had given birth than among nulliparous women, indicating that pregnancy reduced the risk of meningioma. Only 11 patients of 2,353,894 pregnancies had a meningioma diagnosed during the pregnancy, compared to 27 expected, and delayed diagnosis did not explain the lower risk among parous women compared to nulliparous.

The major strength of the present study is the large, comprehensive cohort design with complete follow-up. All women of childbearing age in Sweden were included during a long study period. They were followed through nationwide, high quality Swedish healthcare data and population registers which allow identification and long-term follow-up of all persons. From a methodological perspective, these data are stronger than those provided by a case-control design, as the latter may suffer from selection bias caused by non-participation among cases and controls. The present findings contradict the results of case-control studies by Wigertz et al. and Claus et al.^5,13^ In the former, participation was 75% among cases and 52% among controls, while in the latter the corresponding numbers were 65% and 52%^5,13^. Our results transparently represent large population data, and arguably provide the best epidemiological estimate of meningioma incidence related to pregnancy currently available.

One other factor that may affect detection of tumors during pregnancy is the fact that most meningiomas grow slowly and cause insignificant and non-specific symptoms over a long time^29–32^. It is possible that a pregnancy attenuates the focus on the mother’s health, causing neglect of mild symptoms and diagnostic delay in comparison to nulliparous women. In addition, radiological examinations during pregnancy are avoided unless indications are considered very strong. However, diagnostic delays cannot account for the low number of meningiomas diagnosed during and after pregnancy, since the low number of meningiomas diagnosed during pregnancywas not compensated by higher than expected numbers during the first-year post-partum: forty cases were observed compared to the expected 38.6.

Elucidating the mechanistic relationship between meningioma development and sex steroids is complicated by the diverse effects of estrogen and progesterone. The net results include cellular proliferation, as well as differentiation and apoptosis^33^. Meningioma and breast cancer are associated and may have similar causes. Hormone-receptor positive breast cancer is inversely associated with childbirth, while hormonal replacement therapy, the use of oral contraceptives and a long interval between menarche and first birth had a strong correlation with breast cancer^34–36^. In contrast, parity and oral progestin-containing contraceptives protect against endometrial- and ovarian cancers^33,37–39^.

Our findings, and previous literature on pregnancy associated meningioma growth, could be compatible, provided that pregnancy-associated meningiomas comprise a biologically distinguishable small subgroup of meningiomas. Meningiomas detected during pregnancy have unique features that differ from most meningiomas^24,25^. Most are parasellar and chordoid- and clear cell morphology are overrepresented^25^. It is thus probable that a possible subgroup of hormone-dependent pregnancy-related meningiomas was too small to be detected in this study. Recently, the final report based on the French National Health Data System declared an unequivocal causal relation between the progestin cyproterone acetate and meningioma growth in a subgroup of hormonally dependent tumors^14^. The initial reports of mifepristone, an antiprogestogen, therapy for unresectable meningiomas showed opposite effects in the small number of patients treated^40^, and some patients who received therapy with the selective estrogen receptor modulator tamoxifen for breast-cancer appear to have been protected from meningiomas^41,42^. Recently, an increased mutational frequency of PIK3CA, found in several hormonally associated cancers, has been identified in progestin-associated meningiomas^43^. The sum of observations suggests that there are subgroups of meningioma, which may display hormonal dependence and show accelerated growth during pregnancy, while the majority do not.

An alternative explanation for the higher meningioma incidence in nulliparous women could be that nulliparity may be an epiphenomenon linked to poor health. Overall in our dataset, nulliparous women had higher mortality and died at younger ages than parous women. Nulliparity may thus in itself be a result of poorer health^42–47^. Excluding women with genetic syndromes known to be associated with the occurrence of central nervous system tumors did not change our results. There is some evidence that morbidities such as hypertension and diabetes are related to a higher incidence of meningioma^48^, although their relation to parity is unclear.

A limitation in our study is the lack of information about other sources of hormonal alterations in women, such as miscarriages, abortions, and use of exogenous sex hormones. Hormonal flux occurs already during early pregnancy compared to the normal menstrual cycle^18^. Women who were exposed to altered levels of sex hormones during early terminated pregnancies were not classified as “pregnant” in this study and may have been analyzed among “nulliparous” unless they had given birth at another date. Such misclassification would, however, attenuate an association and cannot explain the higher incidence of meningioma in nulliparous women. Thus, the omission of early terminated pregnancies is probably of limited significance for a hypothesis regarding hormonal influence.

The Swedish cancer registry employs a system of dual reporting by pathologists and clinicians and has a complete coverage of all histologically verified meningiomas, detected either at surgery or at autopsy. However, patients with conservatively managed meningiomas are reported only by the clinicians who establish the diagnosis; there is also evidence of underreporting of benign brain tumors to the Swedish cancer registry^49^. Nonetheless, there is no a priori reason to suspect selection bias since indications for surgery during the long follow-up period will not be influenced by a history of pregnancy.

## Conclusion

Our findings showed a decreased risk of meningioma during pregnancy and during follow-up after childbirth compared to nulliparous women in the age group 15–57. These results contradict the belief that hormonal influence during pregnancy increases the detection rate of meningioma.
